# The mitochondrial genome of *Grapsus albolineatus* (Decapoda: Brachyura: Grapsidae) and phylogenetic associations in Brachyura

**DOI:** 10.1038/s41598-022-06080-3

**Published:** 2022-02-08

**Authors:** Jiayin Lü, Liping Xia, Xiaojuan Liu, Yanwen Ma, Jiji Li, Yingying Ye, Baoying Guo

**Affiliations:** 1 National Engineering Research Center for Marine Aquaculture, Zhejiang Ocean University, Zhoushan, 316022 China; 2 Guangdong Provincial Key Laboratory of Marine Biotechnology, Shantou University, Shantou, 515063 Guangdong China

**Keywords:** Phylogenetics, Mitochondrial genome

## Abstract

Complete mitochondrial genomes (mitogenomes) can provide useful information for phylogenetic relationships, gene rearrangement, and evolutionary traits. In this study, we determined the complete mitochondrial DNA sequence of the herbivorous crab *Grapsus albolineatus*. It is a typical metazoan mitochondrial genome. The total size is 15,583 bp, contains the entire set of 37 genes, and has an AT-rich region. Then, 23 of the 37 genes were encoded by the heavy (+) strand while 14 are encoded by the light (−) strand. Compared with the pan-crustacean ground pattern, two tRNA genes (*tRNA-His* and *tRNA-Gln*) were rearranged and the tandem duplication/random loss model was used to explain the observed gene rearrangements. The phylogenetic results showed that all Grapsidae crabs clustered together as a group. Furthermore, the monophyly of each family was well supported, with the exception of Menippidae. In general, the results obtained in this study will contribute to the better understanding of gene rearrangements in Grapsidae crab mitogenomes and provide new insights into the phylogeny of Brachyura.

## Introduction

Brachyura crab is the largest clade in the Decapod crustacean group, with more than 7250 known species, including 98 families of marine, freshwater, and terrestrial habitats, most of which are economically important^1^. However, the phylogenetic relationships among members of Brachyura and their evolutionary origin continue to be controversial due to the high morphological similarity and ecological diversity^2–4^. Initially, Brachyura was divided into Podotremata, Heterotremata, and Thoracotremata^5^. Subsequently, it was segmented into Dromiacea and Eubrachyura (including Thoracotremata, Raninoida, and Heterotremata)^6^. However, the latest classification scheme divides Brachyura into Cyclodorippoida, Eubrachyura, Dromicea, and Raninoida^7,8^. Although the phylogenetic relationship within Brachyura is still uncertain, the current classification system has been recognized by most scholars.

According to WoRMS (http://www.marinespecies.org/), the family Grapsidae has 8 genera and 49 species in total. However, only five species sequences of Grapsidae have been published^4,9–12^. The herbivorous crab (*Grapsus albolineatus*) is one of the marine crustaceans that live on rocky shores which belongs to the phylum Arthropod, subphylum Crustacea, order Decapoda, infraorder Brachyura, clade Thoracotremata, family Grapsidae, genus *Grapsus*. They are mainly distributed in Japan, Hawaii, Australia and China’s Guangdong, Hainan Island, Xisha Islands, Taiwan. So far, most studies of this species have focused on the morphology and growth^13,14^. Although there are few studies on the molecular level, most of them were based on partial mitochondrial and nuclear ribosomal RNA gene sequences^15^.

The mitochondrial genome (mitogenome) of metazoans is usually 14–20 kb in size and encoded with a set of 37 genes, including 13 protein coding genes (*cox1-3*, *cob*, *nad1-6*, *nad4L*, *atp6*, and *atp8*), 2 ribosomal RNA genes (*rrnl* and *rrns*), 22 transport RNA genes (tRNAs), and an AT-rich region (also called control region, CR) which contains some initiation sites for transcription and replication of the genome^16^. Mitochondrial DNA forms a separate unit of genetic information that evolved independently from the nuclear genome. Due to its haploid properties, matrilineal inheritance, limited recombination, and rapid rate of evolution^17^, the mitogenome is increasingly being used in evolutionary and phylogenetic studies. With the rapid development of sequencing technology, next-generation sequencing has become a fast and low-cost method to provide complete mitotic genomes^18^.

Gene rearrangements in the mitogenomes of crabs are relatively common^1,19,20^. So far, several hypotheses have been suggested to help explain gene rearrangements in animal mitogenomes. Recombination model and tandem duplication/random loss (TDRL) model are more commonly accepted. Recombination models are involved in the breaking and reconnecting of DNA strands^21^. The TDRL model assumes that the rearranged gene order occurs via tandem duplications followed by random deletion of certain duplications^22^. This model has been widely used to explain the translocation of genes encoded on the same strand^23^. Model tRNA mis-priming model and the tandem duplication/non-random loss model (TDNL) are less commonly used.

In this study, we successfully sequenced the complete mitogenome of *G. albolineatus* and used existing complete mitogenomes to compare it with other Brachyura species. In addition, a phylogenetic analysis of 70 brachyuran species was conducted based on the nucleotide sequences of 13 PCGs (Protein-coding gene). These results will help us to understand features of the *G. albolineatus* mitogenome and the evolutionary relationships within Brachyura.

## Results and discussion

### Genome structure and composition

**Figure 1:**
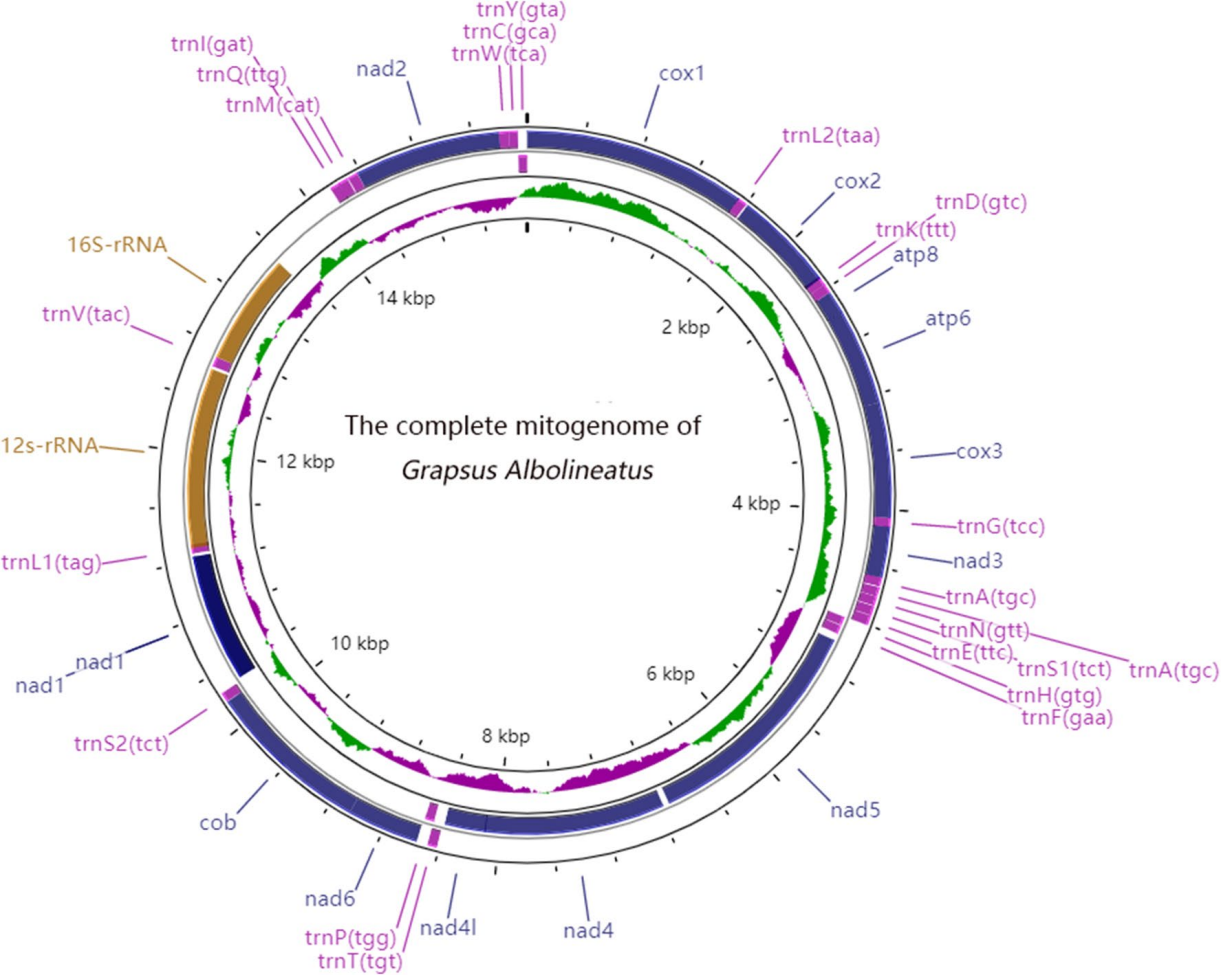
Circular mitogenome map of *Grapsus albolineatus*. Protein coding, ribosomal, and tRNA genes are shown with standard abbreviations. Arrows indicate the orientation of gene transcription. The inner circles show the G–C content and GC-skew, which are plotted as the deviation from the average value of the entire sequence.

The complete mitogenome sequence of *G. albolineatus* is a typical closed-circular molecule of 15,583 bp in size (GenBank accession number MZ262276), which is similar in length to the published Grapsidae mitogenomes^4,9–12^, a size range from 15,406 to 15,920 bp (Table 1). The mitogenome contents of *G. albolineatus* is the same as most other published Brachyura which includes 37 genes, 13 PCGs, 22 tRNAs, and 2 rRNA (*rrnl* and *rrns*), as well as a brief non-coding region, all the genes were identified (Fig. 1, Table 2). Most of the 37 genes are located on the heavy (H-) strand, except 4 PCGs (*ND5*, *ND4*, *ND4L*, *ND1*), 8 tRNAs (*tRNA-Cys*, *Tyr*, *Gln*, *Val*, *Leu*, *Pro*, *Phe*, and *His*), and 2 rRNA which are located on the light (L-) strand (Fig. 1, Table 2). There are 13 regions with overlap in the total *G. albolineatus* mitogenome, with 3 of them more than 10 bp (*trnT* (41 bp), *trnL*_*1*_ (25 bp), and *cox2/trnS*_*2*_ (20 bp)) and the other 10 shorter than 10 bp (*nad4* (7 bp), *atp8* (4 bp), *cox3/atp6/rnK/nad6/trnW* (1 bp), *trnG* (3 bp), and *nad3/nad2* (2 bp)) (Table 2). The *G. albolineatus* mitogenome also contains 328 bp of intergenic spacers located in 17 regions, ranging from 1 to 122 bp (Table 2) and indicating the occurrence of tandem duplications and the deletions of redundant genes. GC-skew of the complete mitogenomes of 6 Grapsidae species were calculated and compared (Tables 3, 4). The nucleotide composition of the *G. albolineatus* mitogenome is A (33.4%), T (34.04%), G (12.02%), and C (20.54%), with a high A–T bias. The A + T (%) content of the mitogenomes was 66.74%. The AT-skew and GC-skew value are calculated for the chosen complete mitogenomes (Table 3). Both AT-skew and GC-skew of the *G. albolineatus* mitogenome are slightly negative, −0.009 and −0.262, informing T’s and C’s are more abundant than A’s and G’s. Similar results were observed for the other selected Grapsidae mitogenomes. In general, the AT-skew and GC-skew of the overall mitogenomes, nucleotide composition, and gene lengths of the *G. albolineatus* were the same as those of the other Grapsidae species^4,9–12^.

**Table 1:** List of Brachyuran species with their GenBank accession numbers.

Superfamily	Family	Species	Size (bp)	Accession.no
Grapsoidea	Grapsidae	*Pachygrapsus marmoratus*	15,406	MF457403.1
		*Grapsus albolineatus*	15,583	MZ262276
		*Metopograpsus frontalis*	15,587	NC_042152.1
		*Metopograpsus quadridentatus*	15,520	MH310445
		*Grapsus tenuicrustatus*	15,858	NC_029724
		*Pachygrapsus crassipes*	15,652	NC_021754
	Sesarmidae	*Parasesarma pictum*	15,611	NC_ 038,066
		*Parasesarma tripectinis*	15,612	NC_030046
		*Perisesarma bidens*	15,641	NC_051868
		*Parasesarma affine*	15,638	NC_ 039,990
		*Chiromantes haematocheir*	15,899	NC_042142.1
		*Sesarma neglectum*	15,920	NC_031851.1
	Varunidae	*Pseudohelice subquadrata*	16,898	MH718959
		*Hemigrapsus penicillatus*	16,486	MG71772.1
		*Varuna yui*	15,915	NC_037155
		*Varuna litterata*	16,378	MF 198,252.1
		*Cyclograpsus intermedius*	16,184	MT621398.1
		*Cyclograpsus granulosus*	16,300	NC_025571
		*Metaplax longipes*	16,424	MF 198,248
		*Eriocheir sinensis*	16,378	KM516908
		*Chasmagnathus convexus*	15,107	NC_052834.1
		*Gecarcoidea lalandii*	15,575	NC_057475.1
		*Gecarcoidea natalis*	15,545	NC_039811.2
	Xenograpsidae	*Xenograpsus ngatama*	15,798	EU727203
		*Xenograpsus testudinatus*	15,798	NC_013480.1
Ocypodoidea	Dotillidae	*Ilyoplax deschampsi*	15,460	NC_020040
	Macrophthalmidae	*Macrophthalmus pacificus*	17,226	NC_046039
		*Macrophthalmus latreillei*	15,747	MW423579
		*Macrophthalmus abbreviatus*	16,322	MN393095
		*Macrophthalmus japonicus*	16,170	NC_030048
		*Scopimera intermedia*	16,252	MW165226
	Mictyridae	*Mictyris longicarpus*	15,548	LN611670
		*Mictyris thailandensis*	15,557	MW697086
	Ocypodidae	*Ocypode ceratophthalmus*	15,564	NC_025324
		*Ocypode stimpsoni*	15,557	NC_046797
		*Austruca lactea*	15,659	NC_042401
		*Cranuca inversa*	15,677	MF457405
		*Tubuca capricornis*	15,629	MF457401
		*Tubuca rosea*	15,643	MN072632
		*Tubuca polita*	15,672	NC_039106
		*Tubuca arcuata*	15,727	MN893258
Bythograeoidea	Bythograeidae	*Gandalfus puia*	15,548	NC_027414
		*Austinograea alayseae*	15,611	KC851803
		*Segonzacia mesatlantica*	15,521	NC_035300
Calappoidea	Calappidae	*Calappa bilineat*	15,606	NC_047195
	Matutidae	*Ashtoret lunaris*	15,807	NC_024435
		*Matuta planipes*	15,751	MK281334
		*Matuta victor*	15,782	NC_05363
Carpilioidea	Carpiliidae	*Carpilius convexus*	15,766	MT780873
		*Carpilius maculatus*	15,761	NC_049030
Eriphioidea	Menippidae	*Myomenippe fornasinii*	15,658	NC_024437
		*Pseudocarcinus gigas*	15,515	AY562127
	Oziidae	*Epixanthus frontalis*	15,993	MF457404
Xanthoidea	Xanthidae	*Etisus anaglyptus*	16,435	NC_042208
		*Etisus dentatus*	15,884	NC_054248
		*Atergatis integerrimus*	15,924	NC_037172
		*Atergatis floridus*	16,180	NC_037201
Majoidea	Oregoniidae	*Chionoecetes japonicus*	15,341	AB735678
	Majidae	*Maja crispata*	16,592	NC_035424.1
		*Maja squinado*	16,598	NC_035425.1
Portunoidea	Geryonidae	*Chaceon granulatus*	16,135	NC_023476.1
		*Chaceon* sp.	16,126	KU507298
	Portunidae	*Thalamita crenata*	15,787	NC_024438
		*Thalamita sima*	15,831	NC_039640
		*Portunus trituberculatus*	16,026	AB093006
		*Portunus gracilimanus*	15,990	NC_040124
		*Charybdis natator*	15,664	MF285241
		*Charybdis japonica*	15,738	FJ460517
		*Charybdis feriata*	15,660	KF386147
Outgroup		*Pagurus nigrofascia*	15,423	NC_042412
		*Pagurus gracilipes*	16,051	LC222534

**Table 2:** Nucleotide composition and skewness of *Grapsus albolineatus* mitochondrial genome.

*G. albolineatus*	A%	T%	G%	C%	(A + T)%	AT-skew	GC-skew	Length (bp)
Mitogenome	33.4	34.04	12.02	20.54	67.44	− 0.009	− 0.262	15,583
*PCGs*	27.44	37.82	16.78	17.96	65.26	− 0.159	− 0.034	11,323
*cox1*	26.90	34.50	16.31	22.29	61.40	− 0.124	− 0.155	1539
*cox2*	30.79	32.77	14.69	21.75	63.56	− 0.031	− 0.194	708
*atp8*	28.93	7.55	45.28	18.24	74.21	0.586	0.426	159
*atp6*	37.05	28.27	12.20	22.47	65.33	0.134	− 0.296	672
*cox3*	28.41	33.71	15.78	22.10	62.12	− 0.085	− 0.167	792
*nad3*	26.84	38.70	22.03	22.03	65.54	− 0.181	0.000	354
*cox3*	29.29	38.30	20.68	11.73	70.30	− 0.133	0.276	1731
*nad5*	27.80	39.61	22.65	9.94	39.61	− 0.175	0.390	1338
*nad4*	27.80	39.61	22.65	9.94	67.41	− 0.175	0.390	1338
*nad4L*	28.71	41.58	21.45	8.25	70.30	− 0.183	0.444	303
*nad6*	23.49	43.37	10.64	22.49	66.87	− 0.297	− 0.358	498
*cob*	26.52	35.51	14.19	23.79	62.03	− 0.145	− 0.253	1135
*nad1*	23.95	41.77	22.57	11.71	65.72	− 0.271	0.317	948
*nad2*	25.62	39.86	10.88	23.64	65.48	− 0.217	− 0.370	1011
tRNAs	35.45	36.09	16.48	11.98	71.54	− 0.009	0.158	1402
rRNAs	36.24	36.33	17.61	9.82	72.57	− 0.001	0.284	2158
AT-rich	45.54	32.09	8.91	13.45	77.63	0.173	− 0.203	617

**Table 3:** Organization of the *Grapsus albolineatus* mitochondrial genome*.*

Gene	Position		Length	Amino acid	Start/stop codon	Anticodon	Intergenic region	Strand
	From	To						
*cox1*	1	1539	1539	513	ATG/TAG		0	H
*trnL2*	1535	1602	68			TAA	10	H
*cox2*	1613	2320	708	236	ATG/TAA		−20	H
*trnK*	2301	2370	70			TTT	−1	H
*trnD*	2370	2433	64			GTC	0	H
*atp8*	2434	2592	159	53	GTG/TAA		−4	H
*atp6*	2589	3260	672	224	ATA/TAA		−1	H
*cox3*	3260	4051	792	264	ATG/TAA		−1	H
*trnG*	4051	4113	63			TCC	−3	H
*nad3*	4111	4464	354	118	ATA/TAA		−2	H
*trnA*	4463	4526	64			TGC	6	H
*trnR*	4533	4596	64			TCG	1	H
*trnN*	4598	4662	65			GTT	4	H
*trnS1*	4667	4733	67			TCT	2	H
*trnE*	4736	4803	68			TTC	3	H
*trnH*	4807	4871	65			GTG	4	L
*trnF*	4876	4940	65			GAA	52	L
*nad5*	4993	6723	1731	577	ATT/TAA		44	L
*nad4*	6768	8105	1338	446	ATG/TAG		−7	L
*nad4L*	8099	8401	303	101	ATG/TAA		5	L
*trnT*	8416	8481	50			TGT	−41	H
*trnP*	8482	8550	69			TGG	8	L
*nad6*	8559	9056	498	166	ATT/TAA		−1	H
*cob*	9056	10,190	1134	378	ATG/TAA		0	H
*trnS2*	10,191	10,258	927	309		TCT	0	H
*nad1*	10,286	11,233	948	316	ATT/TAA		23	L
*trnL1*	11,257	11,323	67			TAG	−25	L
*rrnL*	11,299	12,629	1331				21	L
*trnV*	12,651	12,723	73			TAC	0	L
*rrnS*	12,724	13,550	827				122	L
*CR*	13,551	14,167	617				0	H
*trnI*	14,168	14,234	155			GAT	70	H
*trnQ*	14,232	14,300	69			TTG	7	L
*trnM*	14,308	14,378	71			CAT	0	H
*nad2*	14,379	15,389	1011	367	ATT/TAG		−2	H
*trnW*	15,388	15,456	69			TCA	−1	H
*trnC*	15,456	15,519	64			GCA	0	L
*trnY*	15,520	15,583	64			GTA	0	L

**Table 4:** Nucleoride composition in regions of the mitogenomes of six Grapsidae species.

Species	Total size	Complete mitogenome						
		A	T	G	C	A + T%	AT-skew	GC-skew
*Pachygrapsus crassipes*	15,652	36.61	38.2	10.06	15.13	74.81	− 0.021	− 0.201
*Pachygrapsus marmoratus*	15,406	31.4	36.99	12.13	19.49	68.38	− 0.082	− 0.233
*Grapsus albolineatus*	15,583	33.4	34.04	12.02	20.54	67.44	− 0.009	− 0.262
*Grapsus tenuicrustatus*	15,858	31.92	33.11	12.13	22.85	65.03	− 0.018	− 0.306
*Metopograpsus frontalis*	15,587	32.77	36.95	11.01	19.27	69.72	− 0.060	− 0.273
*Metopograpsus quadridentatus*	15,520	34.25	26.01	10.21	19.53	70.26	0.137	− 0.313
		PCGs						
*Pachygrapsus crassipes*	11,160	25.89	38.99	17.26	17.87	64.87	− 0.202	− 0.017
*Pachygrapsus marmoratus*	11,178	26.79	40.39	16.62	16.69	67.19	− 0.202	− 0.002
*Grapsus albolineatus*	11,323	27.44	37.82	16.78	17.96	65.26	− 0.159	− 0.034
*Grapsus tenuicrustatus*	11,463	25.83	37.59	17.34	19.24	63.42	− 0.185	− 0.052
*Metopograpsus frontalis*	11,217	27.79	40.31	15.94	15.96	68.10%	− 0.184	− 0.001
*Metopograpsus quadridentatus*	11,125	28.3	40.25	15.49	15.96	68.55	− 0.174	− 0.015
		tRNAs						
*Pachygrapsus crassipes*	1,485	35.15	35.29	16.5	13.06	70.44	− 0.002	0.116
*Pachygrapsus marmoratus*	1,463	35.82	35.41	16.13	12.65	71.22	0.006	0.121
*Grapsus albolineatus*	1402	35.45	36.09	16.48	11.98	71.54	− 0.009	0.158
*Grapsus tenuicrustatus*	1487	34.97	35.17	16.75	13.11	70.14	− 0.003	0.122
*Metopograpsus frontalis*	1467	36.26	36.74	14.52	12.47	73.01	− 0.007	0.076
*Metopograpsus quadridentatus*	1474	35.41	37.31	15.54	11.74	72.73	− 0.026	0.139
		rRNAs						
*Pachygrapsus crassipes*	2228	37.52	32.94	19.12	10.41	70.47	0.065	0.295
*Pachygrapsus marmoratus*	2187	38.23	34.2	17.88	9.69	72.43	0.056	0.297
*Grapsus albolineatus*	2158	36.24	36.33	17.61	9.82	72.57	− 0.001	0.284
*Grapsus tenuicrustatus*	2239	35.57	34.03	21.04	9.56	69.41	0.022	0.375
*Metopograpsus frontalis*	2172	39.73	34.16	17.22	8.89	73.9	0.075	0.319
*Metopograpsus quadridentatus*	1990	38.89	35.13	17.13	8.09	74.02	0.051	0.358

### PCGs and codon usage

The initial and terminal codons of all PCGs of *G. albolineatus* are listed in Table 2. *G. albolineatus* has 13 PCGs in the typical order found in Brachyuran species, containing 7 NADH dehydrogenase (*nad1-nad6*, *nad4L*), 3 cytochrome c-oxidases (*cox1–cox3*), two ATPases (*atp6*, *atp8*), and cytochrome b (*cob*). The total length of the 13 PCGs is 11,323 bp. The length of the 13 PCGs range from 303 to 1371 bp (Tables 2, 3).

**Figure 2:**
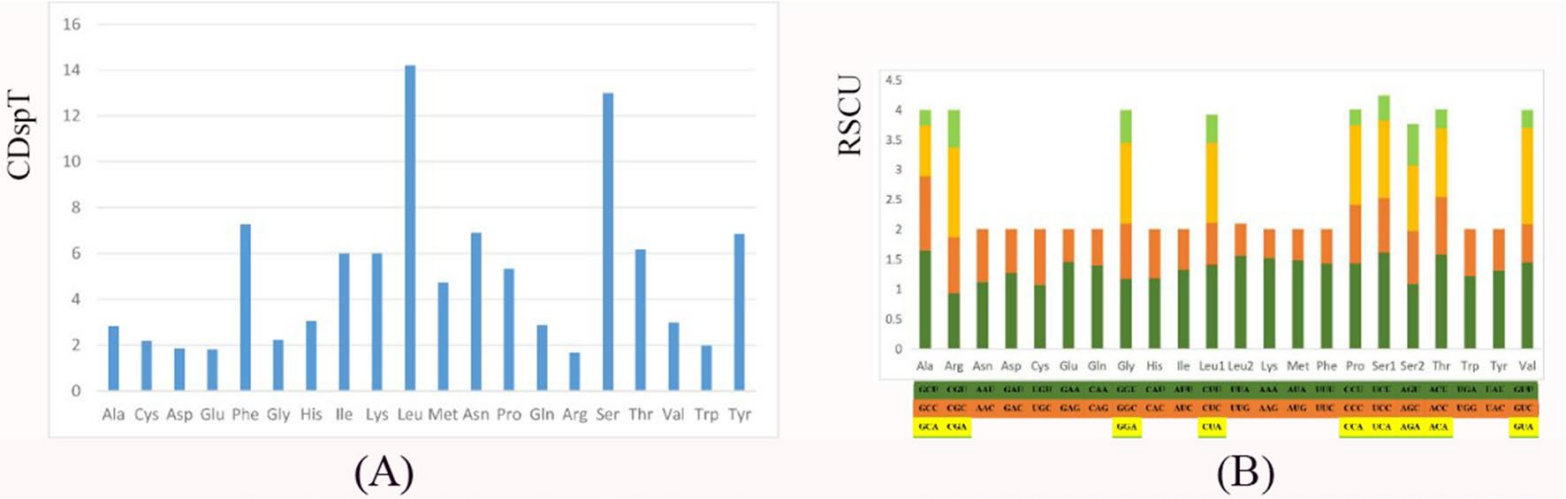
Codon usage patterns in the mitogenome of *Grapsus albolineatus* CDspT, codons per thousand codons. Codon families are provided on the x-axis (**A**), and the relative synonymous codon usage (RSCU) (**B**).

The average A + T content is 65.26%, ranging from 39.63% (ND5) to 74.21% (ATP8) (Table 3). The AT-skew and GC-skew are −0.159 and −0.034, respectively (Table 3). All of the PCGs are initiated by the start codon ATN (ATT, ATG, and ATC), except ATP8 (GTG). The majority of the PCGs are terminated with TAA, whereas the other three PCGs (*cox1*, *nad1*, and *nad2*) use TAG as the stop condon (Table 2). The most frequently used amion acid in *G. albolineatus* is Leu, and the least common anion acid is Trp (Fig. 2). The relative synonymous codon usage (RSCU) values for *G. albolineatus* of the 13 PCGs are shown in Table 5 and Fig. 2^24^. The three most frequently detected codons are GCU (Ala), UCU (Ser2), and GUA (Val), whereas GCU (Ala) is the least common codon. Based on CDspT and RSCU, comparative analyses showed that the codon usage pattern of *G. albolineatus* is conserved. The codon usage patterns of 13 PCGs are similar to those of other Grapsidae species.

**Table 5:** The codon number and relative synonmous codon usage in the mitochondrial genome of *Grapsus albolineatus.*

Codon	Count	RSCU	Codon	Count	RSCU	Codon	Count	RSCU	Codon	Count	RSCU
UUU(F)	253	1.43	UCU(S)	127	1.6	UAU(Y)	219	1.31	UGU(C)	57	1.07
UUC(F)	102	0.57	UCC(S)	73	0.92	UAC(Y)	115	0.69	UGC(C)	50	0.93
UUA(L)	179	1.55	UCA(S)	103	1.3	UAA(*)	233	1.51	UGA(W)	59	1.22
UUG(L)	62	0.54	UCG(S)	33	0.42	UAG(*)	76	0.49	UGG(W)	38	0.78
CUU(L)	163	1.41	CCU(P)	93	1.43	CAU(H)	88	1.18	CGU(R)	19	0.93
CUC(L)	80	0.69	CCC(P)	63	0.97	CAC(H)	61	0.82	CGC(R)	19	0.93
CUA(L)	156	1.35	CCA(P)	88	1.35	CAA(Q)	99	1.4	CGA(R)	31	1.51
CUG(L)	54	0.47	CCG(P)	17	0.26	CAG(Q)	42	0.6	CGG(R)	13	0.63
AUU(I)	194	1.32	ACU(T)	118	1.57	AAU(N)	189	1.12	AGU(S)	86	1.08
AUC(I)	99	0.68	ACC(T)	72	0.96	AAC(N)	148	0.88	AGC(S)	71	0.89
AUA(M)	172	1.48	ACA(T)	87	1.16	AAA(K)	221	1.51	AGA(S)	87	1.1
AUG(M)	60	0.52	ACG(T)	24	0.32	AAG(K)	72	0.49	AGG(S)	55	0.69
GUU(V)	53	1.45	GCU(A)	57	1.64	GAU(D)	58	1.27	GGU(G)	32	1.17
GUC(V)	23	0.63	GCC(A)	43	1.24	GAC(D)	33	0.73	GGC(G)	25	0.92
GUA(V)	59	1.62	GCA(A)	30	0.86	GAA(E)	65	1.46	GGA(G)	37	1.36
GUG(V)	11	0.3	GCG(A)	9	0.26	GAG(E)	24	0.54	GGG(G)	15	0.55

### Transfer RNAs and ribosomal RNAs

Like most Grapsidae species, *G. albolineatus* mitogenome contains 22 tRNA genes^20,25,26^. Fourteen of them are encoded by the heavy strain (H-) and the rest are encoded by the light strain (L-). In the whole mitogenome, the size of tRNAs range from 50 to 73 bp and have a total length of 1402 bp, with an obvious AT bias (71.54%) (Table 2). The AT-skew and GC-skew are −0.009 and 0.158, respectively, showing a slight bias toward the use of Ts and an apparent bias toward Cs (Table 3).

The 12S and 16S rRNA genes are 1331 and 827 bp, respectively, which are typically separated by *tRNA-Val* (Table 2). These sizes are similar to those of other Grapsidae species^15–19^. The A-T content of rRNAs is 72.57%. The AT-skew and GC-skew are −0.001 and 0.284, respectively, suggesting a slight bias toward the use of Ts and an apparent bias toward Cs (Table 3). As most typical mitogenomes of other crabs, CR is located between 12S rRNA and *tRNA-Ile*. The 617 bp CR is obviously AT biased (77.63%). The AT-skew and GC-skew are 0.173 and −0.203, respectively (Table 3), indicating an obvious bias toward the use of A’s and C’s. The index of substitution saturation (Iss) was measured as an implemention in DAMBE 5 and the GTR substitution model^25^. Iss is for the combined dataset of all PCGs of the 59 Brachyura mitogenomes and was significantly lower (Iss = 0.674) than the critical values (Iss, cSym = 0.859). The genes are not saturated, so the reconstructed phylogeny was reliable.

### Gene rearrangement

**Figure 3:**
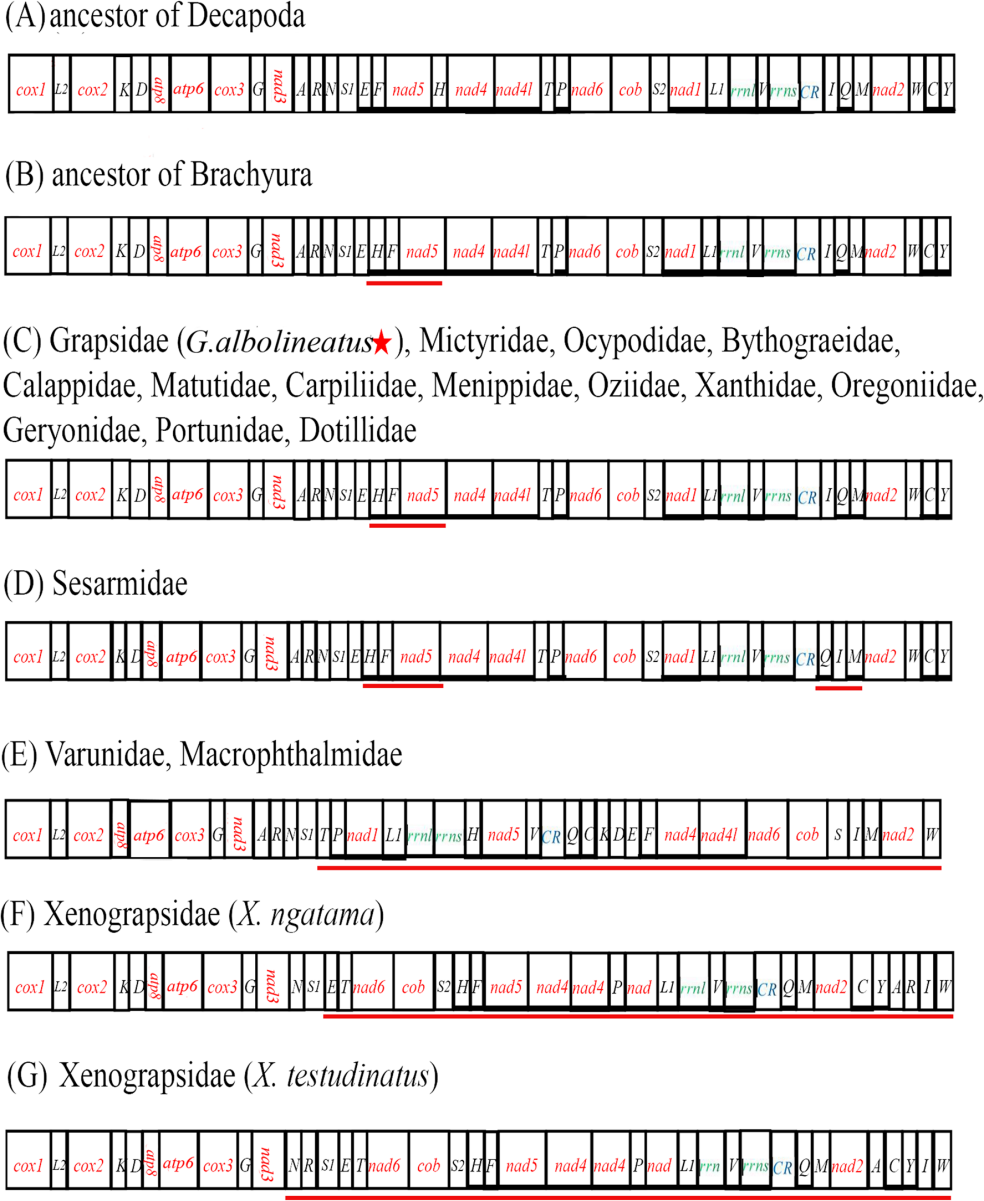
Linear representation of gene arrangements of an (**A**) ancestor of Decapoda, (**B**) ancestor of Brachyura, (**C**) gene arrangement of *Grapsus albolineatus* and 13 familes, (**D**) gene arrangement of Sesarmidae, (**E**) gene arrangement of Varunidae amd Macrophthalmidae, (**F**) gene arrangement of *Xenograpsus testudinatus*, and (**G**) gene arrangement of *Xenograpsus testudinatus*. Gene arrangement of all genes are transcribed from left to right. The green box indicates the duplicated gene. 16S rRNA and 12S rRNA are the large and small ribosomal RNA subunits, respectively. The rearranged gene blocks are underlined and compared with ancestral gene arrangement of Brachyura. The genes encoded on the light strand are highlighted in red.

Mitochondrial gene rearrangement is an important molecular marker and is considered to be an effective tool for studying mitochondrial evolution^26^. A large number of studies and results have shown that gene rearrangements in metazoan mitochondrial genomes are conserved^20^ and the occurrence of gene rearrangements is relatively random and rare^1,19,20,27^. However, it can be used as direct evidence of evolutionary relationships between species^28^. Mapping the gene layout based on the complete mitochondrial sequences of 70 species. Through comparison and analysis with the ancestor of Decapoda (Fig. 3A), we found that *G. albolineatus* and another 5 species from Grapsidae have a *trnH* translocation^4,9–13^, which the *trnH* shifted into *trnE* and *trnF* instead of the usual location between *nad5* and *nad4* (Fig. 3C). It is widely believed that the tandem duplication/random loss model (TDRL) can explain the movement of *trnH*, occur from tandem duplication in the region between *trnE* and *nad4*, followed by deletions of redundant genes producing *trnH-trnF-nad5*. Additionally, 45 species from 14 families (Grapsidae, Mictyridae, Ocypodidae, Bythograeidae, Calappidae, Dotillidae, Matutidae, Menippidae, Oziidae, Xanthidae, Oregoniidae, Geryonidae, Portunidae, and Carpiliidae) had the same gene rearrangement, which are consistent with the ancestral of Brachyura (Fig. 3B). However, the gene order in 4 families (Sesarmidae, Varunidae, Macrophthalmidae, and Xenograpsidae)^30,32^ displayed 4 patterns of gene rearrangements. The family Sesarmidae observed *trnQ* and *trnI* invertred, which has been described in previous studies (Fig. 3D)^3,19,20,33^. The gene order of the Varunidae (Grapsoidea) and Macrophthalmidae (Ocypodoidea) have the same high level rearrangementa (Fig. 3E). It is worth noting that the two families come from two different superfamilies, but they form a sister clade in phylogenetic trees. The gene order of the Xenograpsidae have a more complex rearrangement and such within-genus rearrangements were infrequent^34^ (Fig. 3F,G), which seems to be related to their particular habitat. Xenograpsidae have been found thus far only in shallow-water, volcanically active, and sulphur-rich hydrothermal vents^35^.

### Phylogenetic relationships

**Figure 4:**
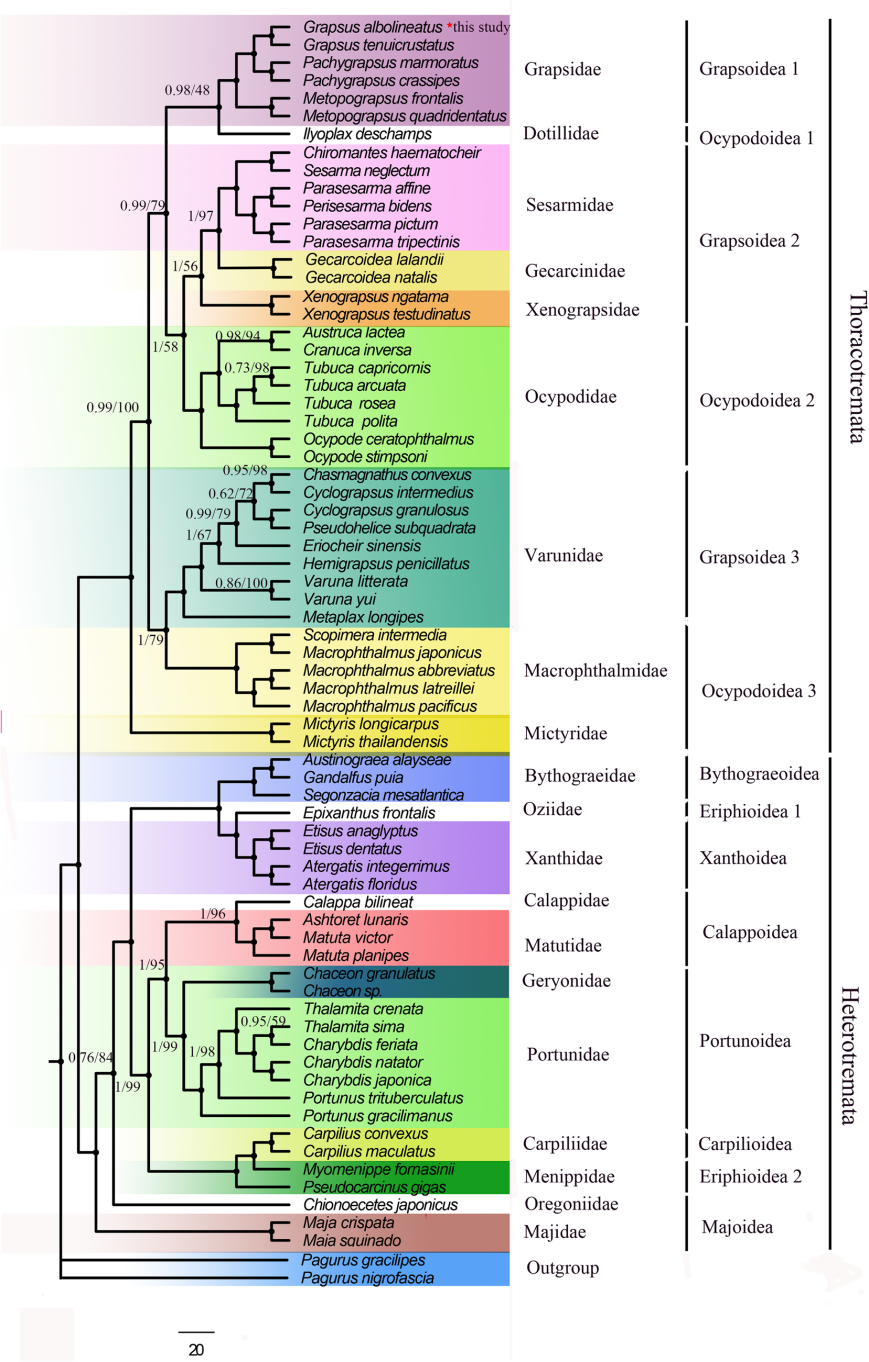
The phylogenetic tree was inferred from the nucleotide sequences of 13 mitogenome PCGs using BI and ML methods. Numbers on branches indicate posterior probability (BI) and bootstrap support (ML). The node marked with a solid citcle indicates 100 ML bootstrap support (BS) and 100% BI posterior probability (PP).

In the present study, the phylogenetic relationships were analyzed based on the sequences of the 13 PCGs to clarify the relationships in Brachyura. *G. albolineatus* and other 68 known brachyuran specie were analyzed, with *P. nigrofascia* and *P. gracilipes* as outgroups. The two phylogenetic trees (Maximum Likelihood (ML) tree and Bayesian Inference (BI) tree) resulted in identical topological structuring with different supporting value. Then, only one topology (ML) with both support values was presented displayed (Fig. 4). Both trees showed that all the species of Grapsidae clustered together as a solid monophyletic group and consist of three sister clades ((*Grapsus* + *Pachygrapsus*) + *Metapograpsus*). It is obvious that *G. albolineatus* had the closest relationship with *G. tenuicrustatus*, and that these two species form a sister clade with high support values (BI posterior probabilities PP = 1, ML bootstrap BP = 100), constituting a *Grapsus* group. However, recent molecular studies, including our dataset, have not reached an agreement about closest relatives in Grapsidae. Our phylogenetic tree showed that Grapsidae and Dotillidae form a sister clade, which was in concordance with Wang et al.^10^. While Wang et al. and Ng, N. K. et al. found that Grapsidae do not have any close relatives^9,35^, Li et al.^36^ found that Grapsidae and Ocypodidae form a sister clade.

Among the 21 families included in our phylogenetic tree, except Menippidae, each family in the tree forms a monophyletic clade with high nodal support values. At a higher level of classification, most Brachyura superfamilies were found to be monophyletic, except Ocypodoidea, Grapsoidea and Eriphioidea, which is in line with previous studies^9,10,37^. It showed that Grapsidea was divided into three clades (((Seasamidae + Gecarcinidae + Xengrapsidae) + Grapsidae) + Varunidae), Ocypodoidea was divided in three clades ((Ocypodidae + Dotillidae) + Macrophthalmidae + Mictyrisae) and Eriphioidea was divided into two clades (Oziidae + Menippidae). Within Thoracotremata, the superfamilies Ocypodoidea and Grapsoidea supported paraphletic and 9 families showed the following relayionship: ((((Seasamidae + Gecarcinidae) + Xengrapsidae) + Ocypodidae) + (Grapsidae + Dotillidae) + (Varunidae + Macrophthalmidae) + Mictyrisae) (Fig. 4).

The main phylogenetic structure of our tree is consistent with previous results, but some controversial findings were observed. Here, the families Macrophthalmidae and Varunidae were grouped into one clade, and Mictyridae as basal group which supports the previous findings revealed in Wang et al. and Zhang et al.^9,33^. However, previous researchers revealed that Macrophthalmidae and Varunidae were grouped into one clade, then into another clade with Varunidae ((Macrophthalmidae + Varunidae) + Mictyridae)^38,39^, which was conflict with our results. The classification of Grapsoidea and Ocypodoidea has long been controversial. Previous studies based on morphological characteristics considered them to be monophyletic branches. However, an increasing number of molecular studies, including ours, challenge the inconsistent views on the traditional classification system that are put forward. Although the polyphyly of Grapsoidea, Ocypodoidea, and Eriphioidea is well supported, the phylogenetic relationships of these superfamilies need to be further analyzed by integrating additional molecular data^32–36^. Previous studies on mitochondrial phylogeny have confirmed the importance of mitochondrial genomic data in elucidating the Grapsidae phylogeny^13,19^. On the contrary, many families contained only one representative, which may produce unstable phylogenetic relationships. Therefore, it is necessary to perform further mitogenome sequence studies to obtain a more comprehensive taxon sampling and understand the phylogeny and evolution of Grapsidae.

## Materials and methods

### Sampling and DNA extraction

A specimen of *G. albolineatus* was collected from Yangjiang, Guangdong Province, China (21°28′45″ N, 111°16′35″ E). The specimen was immediately preserved in absolute ethanol after collection and then stored at −20 °C. This specimen was identified by morphology and fresh tissues were dissected from the operculum and preserved in absolute ethanol before DNA extraction. The total genomic DNA was extracted using the salt-extraction procedure with a slight modification^40^ and stored at −20 °C.

### Genome sequencing, assembly, and annotation

The mitogenomes of *G. albolineatus* was sequenced by Origin gene Co. Ltd., Shanghai, China and was sequenced on the Illumina HiSeq X Ten platform. HiSeq X Ten libraries with an insert size of 300–500 bp were generated from the genomic DNA. About 10 Gb of raw data was generated for each library. Low-quality reads, adapters, and sequences with high “N” ratios and length less than 25 bp were removed. The clean reads were assembled using the software NOVOPlasty (https://github.com/ndierckx/NOVOPlasty)^42^, annotated, and manually corrected on the basis of the complete mitogenome sets assembled de novo by using MITOS tools (http://mitos2.bioinf.uni-leipzig.de/index.py)^43^. To confirm the correct sequences, we compared the assembled mitochondrial genes with those of other *Grapsus* species and identified the mitogenomic sequences by checking the *cox1* barcode sequence with NCBI BLAST^43^. The abnormal start and stop codons were determined by comparing them with the start and stop codons of other marine gastropods. Then, the reads were reconstructed using the de novo assembly program. The complete mtDNA was annotated using the software Sequin version 16.0 (https://trace.ncbi.nlm.nih.gov/Traces/sra). The mitogenome map of the *G. albolineatus* was drawn using the online tool CGView Server (http://cgview.ca/)^45^. The secondary structures predicted of the tRNA genes were plotted by using MITOS Web Server. The relative synonymous codon usage (RSCU) values and substitution saturation for the 13 PCGs, calculated by DAMBE 5^45^, were analyzed with MEGA 7^46^. The GC-skews and AT-skews were used to determine the base compositional difference and strand asymmetry among the samples. According to the following formulas^46^, composition skew values were calculated as AT-skew = A − T/A + T and GC skew = G − C/G + C. Substitution saturation for the 13 PCGs was calculated by DAMBE 5^45^.

### Phylogenetic analysis

The phylogenetic relationships within Brachyura were reconstructed using the sequences of the 13 PCGs of a total of 57 complete mitogenome sequences downloaded from the GenBank database (https://www.ncbi.nlm.nih.gov/genbank/) and adding two species of Paguridae to serve as the outgroup (Table 1). The phylogenetic relationships were analyzed with Maximum Likelihood (ML) by using IQ-TREE 1.6.2 and Bayesian Inference (BI) methods in MrBayes 3.2 version program^47–49^. The ML analysis was inferred with 1000 ultrafast likelihood bootstrap replicates by using IQ-TREE 1.6.2. The best-fit model for each partition was GTR + F + R6, selected according to the Bayesian information criterion (BIC). BI was performed in MrBayes 3.2, and the best-fit evolutionary models were determined using MrMTgui^50^. MrMTgui was used to associate PAUP, ModelTest, and MrModelTest across platforms. MrBayes settings for the best-fit model (GTR + I + G) were selected by Akaike Information Criterion (AIC) in MrModelTest 2.3^51,52^. The Bayesian phylogenetic analyses were performed using the parameter values estimated with the commands in MrModelTest or ModelTest (nst = 6, rates = invgamma)^53^. With three hot chains and one cold chain, they were run simultaneously twice by Markov Chain Monte Carlo (MCMC) sampling, and the posterior distribution was estimated. The MCMC chains were set for 2,000,000 generations and sampled every 1000 steps, with a relative burn-in of 25%. The convergence of the independent runs was evaluated by mean standard deviation of the split frequencies (< 0.01). The phylogenetic trees were visualized and edited using Figure Tree v1.4.3 software^54^.

## Conclusions

In this study, the mitogenome of *G. albolineatus* was sequenced by next-generation sequencing, thereby generating new mitochondrial data for Grapsidae and confirming its ancestral gene order. The *G. albolineatus* mitogenome is a typical closed-circular molecule including 13 PCGs, 22 tRNA genes, two rRNA genes, and a CR. The AT-skew and GC-skew are both negative in the mitogenome of *G. albolineatus*, showing an obvious bias towards the use of T’s and C’s, consistent with published findings in most Brachyura crabs. *G. albolineatus* exhibits a novel gene rearrangement, which is similar to *G. tenuicrustatus*, *P. crassipes*, *P. marmoratu*, *M. frontalis*, and *M. quadridentatus*. Compared with the pan-crustacean ground pattern, the *trnH* of *G. albolineatus* shifted into *trnE* and *trnF* instead of the usual location between *nad5* and *nad4*. By adding 62 Brachyura mitochondrial genomes, rearrangement and the phylogeny of Brachyura was reanalyzed. The phylogenetic analyses indicated that *G. albolineatus* has close relationships with *G. tenuicrustatus*, *P. crassipesand*, *P. marmoratu*, *M. frontalis*, and *M. quadridentatus*, belonging to Grapsoidea, part of the Grapsidae family.

## Data Availability

The complete mitogenome of *Grapsus albolineatus* has been submitted to GenBank under the accession number of MZ262276. The data that support the finding of this study are openly available in Microsoft OneDrive at https://1drv.ms/u/s!Apz_mHDHDJqiUHXhxzoLR0_NEHf?e=u7Ne8W.
